# The association of triglyceride-glucose index and related indices with all-cause and cardiovascular mortality in biologically aging populations

**DOI:** 10.1097/MD.0000000000046036

**Published:** 2025-11-21

**Authors:** Meng-Qun Cheng, Zhong-Ping Bai, Gao Song, Cai-Qiong Zhang, Xing Liu, Rong Li

**Affiliations:** a Department of Reproductive Medicine, Puer People’s Hospital, Pu’er, China; b Department of Pharmacy, Puer People’s Hospital, Pu’er, China; c Department of Pharmacy, The Third People’s Hospital of Kunming/Yunnan Clinical Center for Infectious Diseases, Kunming, China.

**Keywords:** all-cause mortality, biological aging, cardiovascular mortality, KDM biological age, phenotypic age, TyG index, TyG-BMI index, TyG-WHtR index

## Abstract

The relationship between the triglyceride-glucose index (TyG) and all-cause and cardiovascular mortality in biological aging (BA) populations remains unclear. This study aimed to investigate the role of TyG and its derived indices (TyG-Body Mass Index [TyG-BMI] and TyG-Waist-to-Height Ratio [TyG-WHtR]) as potential biomarkers of mortality risk in biologically aging populations. This study included participants from the National Health and Nutrition Examination Survey database between 1999 and 2018 and constructed 3 metabolism-related indices: TyG, TyG-BMI, and TyG-WHtR. Kaplan–Meier survival curves, Cox regression analysis, trend tests, and restricted cubic splines were used to assess the relationship between TyG-related indices and all-cause and cardiovascular mortality in BA populations (including Klemera–Doubal biological age acceleration [KDM-BA] and phenotypic age acceleration [PA]). Subgroup and interaction analyses were performed. Survival analysis showed that, in the KDM-BA population, higher quartiles of TyG, TyG-BMI, and TyG-WHtR were significantly associated with worse survival outcomes. In the PA population, higher TyG and TyG-WHtR levels also indicated worse survival (*P* < .001 for both), but no significant difference was found for TyG-BMI in all-cause mortality (*P* = .313). Fully adjusted Cox regression analysis further confirmed that in the KDM-BA population, TyG and its related indices were significantly associated with both all-cause and cardiovascular mortality hazard ratio (HR range: 1.19–1.34, *P* < .05). In the PA population, TyG was significantly associated with both types of mortality (HR range: 1.16–1.19, *P* < .05), whereas TyG-WHtR was only associated with cardiovascular mortality (HR = 1.26, *P* = .022). TyG-BMI was not significantly associated with either type of mortality. Nonlinear analysis revealed a significant dose-response relationship between TyG and mortality in both populations, with the mortality risk significantly increasing above a certain threshold (approximately 8.1). In the PA population, TyG-BMI and TyG-WHtR showed significant nonlinear relationships with all-cause mortality. Specifically, when TyG-BMI exceeded 215.145, the risk of death increased significantly (HR = 1.00, *P* = .031, nonlinear *P* < .001), and when TyG-WHtR exceeded 5.034, the risk of death also increased significantly (HR = 1.20, *P* < .001, nonlinear *P* = .004). Subgroup analysis showed that the association between TyG and its related indices and mortality risk was stronger in individuals aged < 65 years and those with higher BA (e.g., KDM-BA and PA ≥ 5). Medication use had a limited effect on these associations, with only minor enhancements observed in some subgroups. This study underscores the significance of TyG and its related indices (TyG-BMI and TyG-WHtR) as key indicators of all-cause and cardiovascular mortality in biologically aging populations. Notably, strong associations were found in the KDM-BA cohort, particularly among individuals aged < 65 years and those with advanced BA. These findings suggest that TyG and its related indices may play a crucial role in the long-term management of biologically aging populations and serve as valuable biomarkers for identifying high-risk individuals. This further highlights the importance of metabolic dysregulation in the aging process and its contribution to an increased mortality risk.

## 1. Introduction

Biological age refers to the gradual decline of physiological functions over time, and, compared to chronological age (CA), more accurately reflects an individual’s health status and aging level.^[1,2]^ With the global aging population and the advancement of precision medicine, biological age has gained increasing attention as an important tool for predicting disease risk and guiding interventions.^[3,4]^ Studies have shown that individuals of the same CA can exhibit significant physiological differences, emphasizing the importance of identifying quantifiable and predictive biomarkers for personalized health assessments and aging interventions.^[5–7]^ Various methods for measuring biological age have been developed, including clinical indicators, molecular biomarkers, and composite scoring systems, such as homeostatic dysregulation,^[8]^ the Klemera–Doubal method (KDM),^[9]^ phenotypic age,^[10]^ and allostatic load,^[11]^ all of which have demonstrated good performance in predicting mortality risk, age-related diseases, and functional decline.

Cardiovascular diseases (CVDs) are the leading cause of death worldwide, causing over 17 million deaths annually. Aging, as a core risk factor, exacerbates cardiovascular damage by regulating key pathways. Insulin resistance (IR), a key metabolic disorder in aging, exacerbates mitochondrial dysfunction by inhibiting AMP-activated protein kinase (AMPK) and weakens the antioxidant effect of nuclear factor erythroid 2-related factor 2 (Nrf2). This forms a “Aging-IR-Pathway Dysfunction” vicious cycle, accelerating the progression of CVDs.^[12–15]^ IR induces chronic hyperinsulinemia, enhances oxidative stress, and promotes inflammation, thereby accelerating the aging process.^[16]^ In recent years, the triglyceride glucose index (TyG), a simple and stable alternative marker of IR based on fasting triglyceride (TG) and glucose levels, has received widespread attention.^[17]^ Compared to traditional methods, such as the glucose clamp test or homeostasis model assessment of IR, TyG offers greater convenience and clinical accessibility, particularly for large-scale epidemiological studies.^[18]^ Additionally, its derived indices, TyG-body mass index (TyG-BMI) and TyG-waist-to-height ratio (TyG-WHtR), which incorporate an individual’s obesity status, have been shown to be closely associated with various metabolic diseases.^[19,20]^ However, the performance of the TyG and its derivatives in the context of biological aging (BA) – particularly their distribution across distinct BA subtypes (e.g., Klemera–Doubal biological age acceleration [KDM-BA] and phenotypic age [PhenoAge]) and their associations with mortality outcomes – has not been systematically explored.

Given the potential central role of IR in the aging process and related mortality risks, there is an urgent need to identify quantifiable predictive markers reflecting metabolic status that are applicable to biologically aged populations. Therefore, utilizing large-scale representative data from the U.S. National Health and Nutrition Examination Survey(NHANES) database, this study aimed to comprehensively evaluate the distribution patterns of TyG and its derived indices in BA populations and explore their association with all-cause and cardiovascular mortality risks. This will deepen our understanding of the mechanisms of metabolic disturbances in the aging process and provide theoretical and practical tools for early identification and intervention in high-risk populations.

## 2. Methods

### 2.1. Data source

The data for this study were sourced from the NHANES database, covering the period from 1999 to 2018. The NHANES employs a complex, stratified, multi-stage probability sampling design to collect nationally representative health and nutrition data from the civilian population of the United States. This study adhered to the ethical principles outlined in the Declaration of Helsinki and was approved by the National Center for Health Statistics Institutional Review Board. Written informed consent was obtained from all the participants. Detailed information on the study design and data can be accessed from the official website (https://www.cdc.gov/nchs/nhanes/).

### 2.2. Participants selection

**Figure 1.**
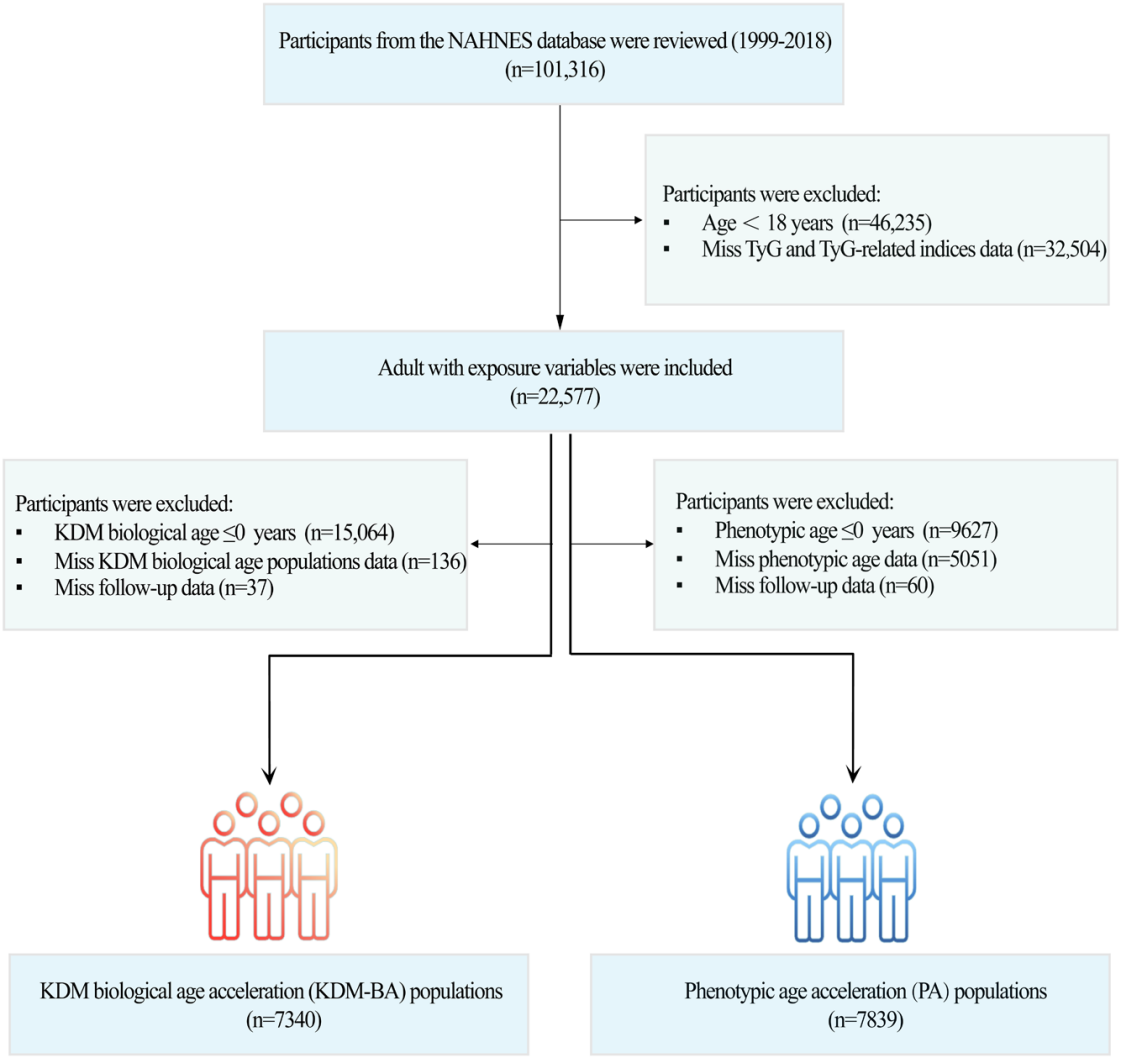
Participant selection process in the present study. A total of 101,316 participants from ten cycles of interviews conducted between 1999 and 2018 were reviewed. Ultimately, 7340 participants with KDM-BA and 7839 participants with PA were included. KDM-BA = Klemera–Doubal biological age acceleration, NHANES = National Health and Nutrition Examination Survey, PA = phenotypic age acceleration, TyG = triglyceride-glucose index.

This study utilized data from ten NHANES cycles (1999–2018), initially comprising 101,316 participants (Fig. 1). Exclusion criteria were applied sequentially: individuals aged < 20 years (n = 46,235) and those with a missing TyG and related exposure variables (n = 32,504) were excluded, yielding 22,577 eligible adults with complete data. For the KDM-BA subgroup, additional exclusions included participants with KDM-BA ≤ 0 years (n = 15,064), missing KDM-BA data (n = 136), and lost to follow-up (n = 37), resulting in a final cohort of 7340. Similarly, for the phenotypic age acceleration (PA) cohort, exclusions comprised individuals with PA ≤ 0 years (n = 9627), missing PA data (n = 5051), or lost to follow-up (n = 60), leaving 7839 participants for the analysis.

### 2.3. Definitions of TyG and TyG-related indices

The TyG is a widely used composite measure that combines TG and fasting blood glucose (FBG) levels to assess IR. This index has been recognized as a reliable and efficient surrogate marker of IR. The TyG, along with its related indices, was calculated using the following formula:


$$\begin{array}{l} TyG=ln[TG(mg/dL)\times FBG(mg/dL)/2] \end{array}$$

$$\begin{array}{l} TyG\text{-}BMI=TyG\times BodyMassIndex(BMI) \end{array}$$

$$\begin{array}{l} TyG\text{-}WHtR=TyG\times Waist\text{-}to\text{-}HeightRatio(WHtR) \end{array}$$

To further analyze the associations, participants were stratified into 4 quartiles (Q1, Q2, Q3, and Q4) based on the distribution of TyG and TyG-related indices, with Q1 serving as the reference group.

### 2.4. Definitions of BA

Biological age is an estimate of an individual’s physiological age, which may differ from that of the CA. It can be assessed using both the KDM method and phenotypic age methods.^[21]^ The KDM biological age was calculated based on 10 biomarkers: systolic blood pressure, albumin, alkaline phosphatase (ALP), blood urea nitrogen (BUN), creatinine, glycated hemoglobin, total cholesterol, lymphocyte percentage, white blood cell count, and mean corpuscular volume. Phenotypic age, on the other hand, was derived from a multivariable analysis based on mortality risk in a reference population using biomarkers such as actual age, albumin, creatinine, glucose, C-reactive protein, lymphocyte percentage, mean corpuscular volume, red blood cell distribution width, ALP, and white blood cell count.

BA is defined as the condition in which the biological age exceeds CA, with biological age acceleration (BA) calculated as the difference between the estimated biological age and CA.^[22]^ In this study, participants with KDM-BA >0 were categorized into the KDM-BA cohort, while those with a PA >0 were assigned to the PA group. The KDM-BA and PA groups were further stratified into 2 categories based on the degree of BA: low (< 5) and high (≥5).

### 2.5. Covariates of interest

Demographic, clinical, and lifestyle data were collected from the participants. Demographic characteristics included age, sex (male or female), race (Non-Hispanic White, Non-Hispanic Black, Mexican American/Other Hispanic, Other Race), education level (less than high school, high school, and above high school), marital status (married or living with a partner, widowed/divorced/separated/never married), and family income-to-poverty ratio (categorized as < 1.3, 1.3–3.5, and >3.5). Clinical variables included the presence of liver disease, cancer, CVD, and chronic kidney disease (CKD). Lifestyle factors such as smoking status (never, ever, current) and alcohol consumption (no, yes) were also considered.

Body mass index (BMI; kg/m²) and laboratory test results, including FBG, TG, BUN, and uric acid (UA) levels, were measured. TyG, TyG-BMI, and TyG-WHtR were also included as metabolic indicators. The use of medications, including antihypertensive agents, statins, and antihyperglycemic agents, was recorded as a categorical variable (yes/no).

### 2.6. Definition of clinical outcomes

Mortality data were obtained from the NHANES Public Use Linked Mortality File, as of December 31, 2019. This file utilizes probabilistic matching to link records with the National Death Index maintained by the National Center for Health Statistics. The cause of death was determined according to the International Statistical Classification of Diseases, 10th Revision, and specific outcomes were reclassified accordingly. All-cause mortality refers to deaths from any cause (010), whereas cardiovascular mortality includes deaths due to heart disease (054–068) and cerebrovascular disease (070).

The primary outcome of this study was the all-cause mortality in the BA population. The secondary outcome was cardiovascular mortality.

## 3. Statistical analysis

All analyses in this study incorporated sample weights, clustering, and stratification to estimate appropriate variance and ensure national. To assess the normality of continuous variables, we primarily used histograms to inspect the distribution of each variable visually. Variable. A normal distribution was considered when the histogram showed a bell shape. As shown in Figures S1 and S2, Supplemental Digital Content, https://links.lww.com/MD/Q724, the continuous variables age, alanine aminotransferase (ALT), aspartate aminotransferase (AST), TG, UA, and FBG showed non-normal distributions. The study population was grouped according to quartiles (Q1–Q4) of TyG and its related indicators. Continuous variables were expressed as median (Q₁, Q₃) if skewed and using a Kruskal–Wallis test, or as mean (standard error) if normally distributed. Comparisons between groups were made using analysis of variance. Categorical variables were expressed as numerical values and weighted percentages (n [weighted %]) using the chi-square test or Fisher exact test, and differences were considered statistically significant at *P* < .05. All analyses adhered to the STROCSS 2024 guidelines (see Table S1, Supplemental Digital Content, https://links.lww.com/MD/Q724 for detailed missing data).^[23]^

The study utilized both univariate and multivariate Cox proportional hazards regression models to assess the association between TyG and its related indicators and the risk of all-cause and cardiovascular mortality in the BA population. The results are presented as hazard ratios (HR) with 95% confidence intervals (CI). For easier comparison, the exposure variables were standardized into *Z*-scores (i.e., the effect size change per 1 standard deviation [SD] increase). Three regression models were constructed: Model 1, unadjusted; Model 2, adjusted for age, sex, and race; and Model 3, further adjusted for age, cancer, sex, race, education level, marital status, poverty-income ratio, smoking status, drinking status, CVD, CKD, ALT, AST, BUN, and UA levels. To account for multicollinearity, variance inflation factors were calculated to assess multicollinearity; variables with variance inflation factors < 5 were included (BMI was excluded because of its high correlation with TyG-BMI and TyG-WHtR). Detailed data of the multicollinearity tests are provided in Table S2, Supplemental Digital Content, https://links.lww.com/MD/Q724. Kaplan (KM) survival curves were used to illustrate the censoring data and survival pattern differences across different TyG quartiles. To explore the dose–response relationship between TyG-related indicators and all-cause and cardiovascular mortality in the BA population (measured by PA/KDM-BA), restricted cubic spline regression was performed with 4 knots, using the median value of the variable as a reference. When a nonlinear relationship was detected, a recursive approach was used to identify potential inflection points, and two-stage fully adjusted Cox models were employed to characterize the risk associations on both sides of the inflection points. Subgroup analyses were conducted based on age, antihypertensive medication use, statin use, antihyperglycemic agents, and severity of BA. Stratification factors were introduced as potential effect modifiers and interaction terms were included to test for differences in effects between the subgroups. Given that this study was exploratory and aimed to generate hypotheses rather than validate them, no corrections for multiple comparisons were made. All data analyses were performed using R software (version 4.2.3, https://www.r-project.org/).

## 4. Result

### 4.1. Baseline characteristics of the BA populations

The study population was divided into 2 BA groups, The KDM-BA group and the PA group, stratified by survival status. Non-survivors were significantly older than survivors, with median ages of 63.00 years (KDM-BA group) and 66.00 years (PA group) compared to 36.00 years and 42.00 years for survivors, respectively (*P* < .001). Non-survivors also had higher levels of ALT, AST, BUN, TG, UA, and FBG as well as elevated TyG, TyG-BMI, and TyG-WHtR (Table 1).

**Table 1:** Baseline characteristics of the biological aging populations stratified by survival status, weighted for representativeness.

Variable	KDM-BA population (n = 7340)		*P* value	PA population (n = 7839)		*P* value
	Survivor (n = 6233)	Non-survivor (n = 1107)		Survivor (n = 6370)	Non-survivor (n = 1469)	
Age, M (Q₁, Q₃)	36.00 (26.00–49.00)	63.00 (49.00–74.00)	**<.001**	42.00 (31.00–54.00)	66.00 (53.00–75.00)	**<.001**
ALT, M (Q₁, Q₃)	22.00 (16.00–32.00)	20.00 (16.00–28.00)	**.002**	23.00 (16.00–32.00)	21.00 (16.00–28.00)	**<.001**
AST, M (Q₁, Q₃)	22.00 (19.00–27.00)	22.00 (19.00–27.00)	.948	22.00 (18.00–27.00)	23.00 (19.00–29.00)	**.001**
TG (Q₁, Q₃)	116.00 (79.00–173.00)	149.00 (100.00–216.00)	**<.001**	115.00 (78.00–167.00)	132.00 (89.00–198.00)	**<.001**
UA (Q₁, Q₃)	5.60 (4.80–6.60)	6.30 (5.30–7.40)	**<.001**	5.60 (4.70–6.60)	5.90 (4.80–6.90)	**<.001**
FBG (Q₁, Q₃)	98.00 (90.50–107.00)	106.90 (96.00–140.10)	**<.001**	99.90 (92.00–109.00)	108.00 (96.50–128.80)	**<.001**
BUN, mg/dL	14.13 (0.10)	19.72 (0.32)	**<.001**	13.47 (0.10)	16.74 (0.24)	**<.001**
BMI, kg/m²	30.63 (0.14)	30.37 (0.33)	.418	30.61 (0.16)	29.55 (0.24)	**<.001**
TyG	8.73 (0.01)	9.16 (0.03)	**<.001**	8.72 (0.01)	9.05 (0.02)	**<.001**
TyG-BMI	268.69 (1.41)	279.25 (3.40)	**.002**	268.15 (1.54)	268.61 (2.35)	.871
TyG-WHtR	5.27 (0.02)	5.77 (0.05)	**<.001**	5.28 (0.02)	5.60 (0.03)	**<.001**
Antihypertensive use, n%			**<.001**			**<.001**
Yes	1807 (75.84)	676 (90.34)		1818 (82.74)	763 (92.23)	
No	453 (24.16)	59 (9.66)		334 (17.26)	61 (7.77)	
Statin use, n (%)			**<.001**			**<.001**
Yes	1096 (33.71)	346 (59.98)		1317 (43.75)	449 (67.95)	
No	1762 (66.29)	174 (40.02)		1482 (56.25)	165 (32.05)	
Antihyperglycemic agent use, n (%)			**.026**			.311
Yes	609 (49.21)	250 (58.87)		700 (52.73)	276 (56.93)	
No	539 (50.79)	162 (41.13)		596 (47.27)	157 (43.07)	
Liver disease, n (%)			**<.001**			**<.001**
Yes	196 (3.03)	68 (7.08)		230 (3.36)	92 (6.82)	
No	6037 (96.97)	1039 (92.92)		6140 (96.64)	1377 (93.18)	
Cancer, n (%)			**<.001**			**<.001**
Yes	308 (4.95)	206 (18.78)		434 (6.16)	302 (20.85)	
No	5925 (95.05)	901 (81.22)		5936 (93.84)	1167 (79.15)	
Gender, n (%)			**<.001**			**.031**
Male	2804 (45.33)	666 (56.84)		3431 (57.17)	950 (61.09)	
Female	3429 (54.67)	441 (43.16)		2939 (42.83)	519 (38.91)	
Race, n (%)			**<.001**			**<.001**
Non-Hispanic White	1651 (15.61)	208 (8.02)		1926 (16.59)	258 (7.77)	
Non-Hispanic Black	538 (6.34)	43 (4.20)		387 (5.28)	33 (2.77)	
Mexican American/Other Hispanic	2461 (64.08)	584 (74.47)		2349 (62.39)	858 (76.61)	
Other Race	1583 (13.97)	272 (13.31)		1708 (15.75)	320 (12.85)	
Education, n (%)			**<.001**			**<.001**
Less than high school	1533 (16.87)	438 (29.48)		1737 (19.00)	622 (33.19)	
High school	1555 (27.01)	292 (31.18)		1560 (27.11)	353 (27.23)	
Above high school	3141 (56.11)	371 (39.34)		3068 (53.89)	489 (39.58)	
Marital status, n (%)			0.401			**.03**
Married/Living with partner	3554 (59.78)	592 (57.93)		3798 (62.95)	781 (58.72)	
Widowed/Divorced/Separated/Never married	2626 (40.22)	497 (42.07)		2490 (37.05)	660 (41.28)	
PIR, n (%)			**<.001**			**<.001**
<1.3	1859 (24.09)	359 (28.01)		1806 (22.37)	471 (27.51)	
1.3–3.5	2234 (37.50)	443 (43.64)		2285 (37.44)	572 (42.51)	
>3.5	1628 (38.41)	201 (28.35)		1703 (40.19)	283 (29.98)	
Smoking status, n (%)			**<.001**			**<.001**
Never	3559 (55.07)	435 (36.99)		3293 (50.55)	482 (30.78)	
Ever	1219 (21.07)	402 (32.93)		1463 (22.99)	585 (37.02)	
Current	1448 (23.86)	269 (30.08)		1608 (26.46)	401 (32.19)	
Drinking status, n (%)			**.005**			**.008**
No	1445 (24.48)	336 (30.70)		1384 (24.42)	437 (28.71)	
Yes	3582 (75.52)	702 (69.30)		3487 (75.58)	938 (71.29)	
CVD, n (%)			**<.001**			**<.001**
No	5753 (94.89)	723 (71.98)		5796 (93.66)	979 (71.50)	
Yes	432 (5.11)	358 (28.02)		521 (6.34)	452 (28.50)	
CKD, n (%)			**<.001**			**<.001**
No	5083 (86.48)	398 (48.34)		5431 (88.57)	794 (63.58)	
Yes	1125 (13.52)	697 (51.66)		939 (11.43)	675 (36.42)	

Analyses used R 4.3.0. Skewed data: median (Q1, Q3; Kruskal–Wallis test); normal data: mean ± SE (ANOVA); categorical data: n (weighted %; chi-square/Fisher test). The bold value indicates *P* < .05, with specific statistical significance.

ALT = alanine aminotransferase, AST = aspartate aminotransferase, BMI = body mass index, BUN = blood urea nitrogen, CKD = chronic kidney disease, CVD = cardiovascular disease, FBG = fasting blood glucose, KDM-BA = Klemera–Doubal biological age acceleration, LDL-C = low-density lipoprotein cholesterol, PA = phenotypic age acceleration, PIR = poverty income ratio, SE = standard error, TG = triglyceride, TyG = triglyceride-glucose index, TyG-BMI = TyG combined with BMI, TyG-WHTR = TyG combined with waist-to-height ratio, UA = uric acid.

Comorbidities such as CVD, CKD, liver disease, and cancer were more prevalent in non-survivors, along with a higher usage of antihypertensive medications and statins. Sociodemographic factors, such as lower education levels, higher smoking and alcohol consumption rates, and a higher proportion of males, were also more common among non-survivors. These findings highlight the association between BA, health conditions, lifestyle factors, and mortality risk.

### 4.2. Survival patterns of the BA population in different quartile levels of TyG-related indices

**Figure 2.**
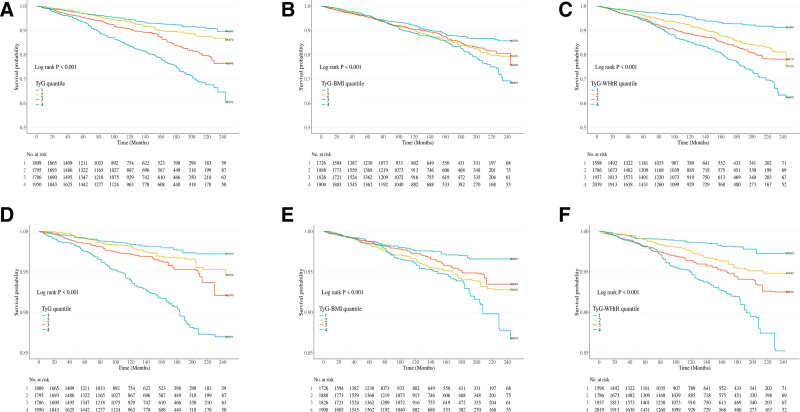
Kaplan–Meier curves show the overall and cause-specific survival probabilities of the KDM-BA population with different quartile levels of TyG, TyG-BMI, and TyG-WHtR indices. (A–C) overall survival with different quartiles of the TyG, TyG-BMI, and TyG-WHtR index; (D–F) cardiovascular-specific survival with different quartiles of the TyG, TyG-BMI, and TyG-WHtR index. BMI = body mass index, KDM-BA = Klemera–Doubal biological age acceleration, TyG = triglyceride-glucose, WHtR = waist-to-height ratio.

**Figure 3.**
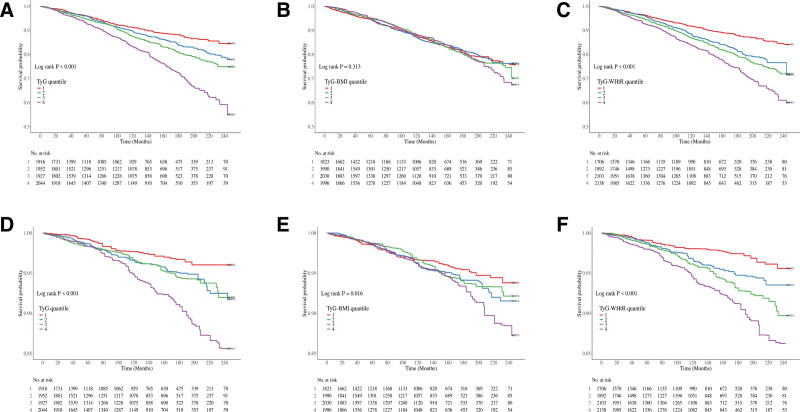
Kaplan–Meier curves show the overall and cause-specific survival probabilities of the PA population with different quartile levels of TyG, TyG-BMI, and TyG-WHtR indices. (A–C) Overall survival with different quartiles of the TyG, TyG-BMI, and TyG-WHtR index; (D–F) cardiovascular-specific survival with different quartiles of the TyG, TyG-BMI, and TyG-WHtR index. BMI = body mass index, PA = phenotypic age acceleration, TyG = triglyceride-glucose, WHtR = waist-to-height ratio.

The survival patterns of the KDM-BA and PA populations were analyzed across different quartiles of TyG-related indices, including TyG, TyG-BMI, and TyG-WHtR. In the KDM-BA population, survival analyses across different quartiles of TyG-related indices (TyG, TyG-BMI, and TyG-WHtR) revealed significant differences, with log-rank tests showing a clear trend of decreased survival in higher quartiles for both all-cause and CVD mortality (*P* < .001 for all indices; Fig. 2A–F). Similarly, in the PA population, KM survival curves indicated that individuals in the higher quartiles of TyG, TyG-BMI, and TyG-WHtR had poorer survival outcomes. Significant differences were observed for TyG (*P* < .001) and TyG-WHtR (*P* < .001; Fig. 3A–F). However, no significant survival differences were found across the TyG-BMI quartiles for all-cause mortality (*P* = .313).

### 4.3. Association of TyG-related indices with all-cause and cardiovascular mortality

**Figure 4.**
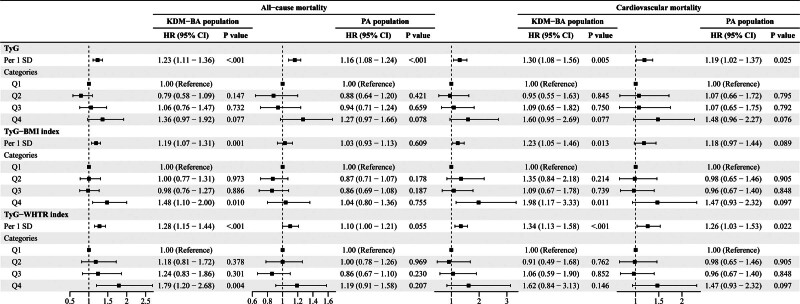
Forest plots of multivariable-adjusted HRs for all-cause and cardiovascular mortality associated with TyG and TyG-derived indices in BA populations. Analysis: weighted Cox proportional hazards models, stratified by survival status and adjusted for adjusted for age, liver disease, cancer, gender, race, education, marital status, PIR, smoking status, drinking, CVD, CKD, ALT, AST, BUN, and UA. Data are presented as HR (95% CI). ALT = alanine aminotransferase, AST = aspartate aminotransferase, BA = biological aging, BUN = blood urea nitrogen, CI = confidence interval, CKD = chronic kidney disease, CVD = cardiovascular diseases, HR = hazard ratio, KDM-BA = Klemera–Doubal biological age acceleration, PA = phenotypic age acceleration, PIR = poverty-income ratio, SD = standard deviation, TyG = triglyceride-glucose index, TyG-BMI = TyG × body mass index, TyG-WHtR = TyG × waist-to-height ratio, UA = uric acid.

In the KDM-BA population, Cox regression analysis (Model 3) showed that TyG, TyG-BMI, and TyG-WHtR were significantly associated with both all-cause and cardiovascular mortalities (Fig. 4). For all-cause mortality, each 1 SD increase in TyG (HR = 1.23, 95% CI: 1.11–1.36, *P* < .001), TyG-BMI (HR = 1.19, 95% CI: 1.07–1.31, *P* = .001), and TyG-WHtR (HR = 1.28, 95% CI: 1.15–1.44, *P* < .001) were associated with an increased risk of mortality. Additionally, the *P*-trend for all-cause mortality across quartiles of TyG (*P* = .003), TyG-BMI (*P* = .006), and TyG-WHtR (*P* < .001) all showed a significant trend towards higher mortality with increasing quartiles (Tables S3–S5, Supplemental Digital Content, https://links.lww.com/MD/Q724). For cardiovascular mortality, TyG (HR = 1.30, 95% CI: 1.08–1.56, *P* = .005), TyG-BMI (HR = 1.23, 95% CI: 1.05–1.46, *P* = .013), and TyG-WHtR (HR = 1.34, 95% CI: 1.13–1.58, *P* < .001) all showed significant associations with increased risk. The *P*-trend for cardiovascular mortality across quartiles was also significant for TyG (*P* = .024), TyG-BMI (*P* = .022), and TyG-WHtR (*P* = .016).

In the PA population, significant associations were found for TyG-related indices in Model 3, although not all the indices showed significant associations (Fig. 4). TyG was significantly associated with an increased risk of all-cause mortality (HR = 1.16, 95% CI: 1.08–1.24, *P* < .001), and the *P*-trend for TyG across quartiles was also significant (*P* = .005). However, neither TyG-BMI (HR = 1.03, 95% CI: 0.93–1.13, *P* = .609) nor TyG-WHtR (HR = 1.10, 95% CI: 1.00–1.21, *P* = .055) was significantly associated with all-cause mortality, and the *P*-trends for these indices were not significant (TyG-BMI *P* = .77, TyG-WHtR *P* = .221). For cardiovascular mortality, TyG was significantly associated with an increased risk (HR = 1.19, 95% CI: 1.02–1.37, *P* = .025), with a significant P-trend across quartiles (*P* = .029). However, while TyG-WHtR (HR = 1.26, 95% CI: 1.03–1.53, *P* = .022) was significantly associated with cardiovascular mortality, TyG-BMI showed no significant association (HR = 1.18, 95% CI: 0.97–1.44, *P* = .089) and the *P*-trend for TyG-BMI was not significant (*P* = .114).

### 4.4. Nonlinear trends of TyG-related indices with mortality of BA population

**Figure 5.**
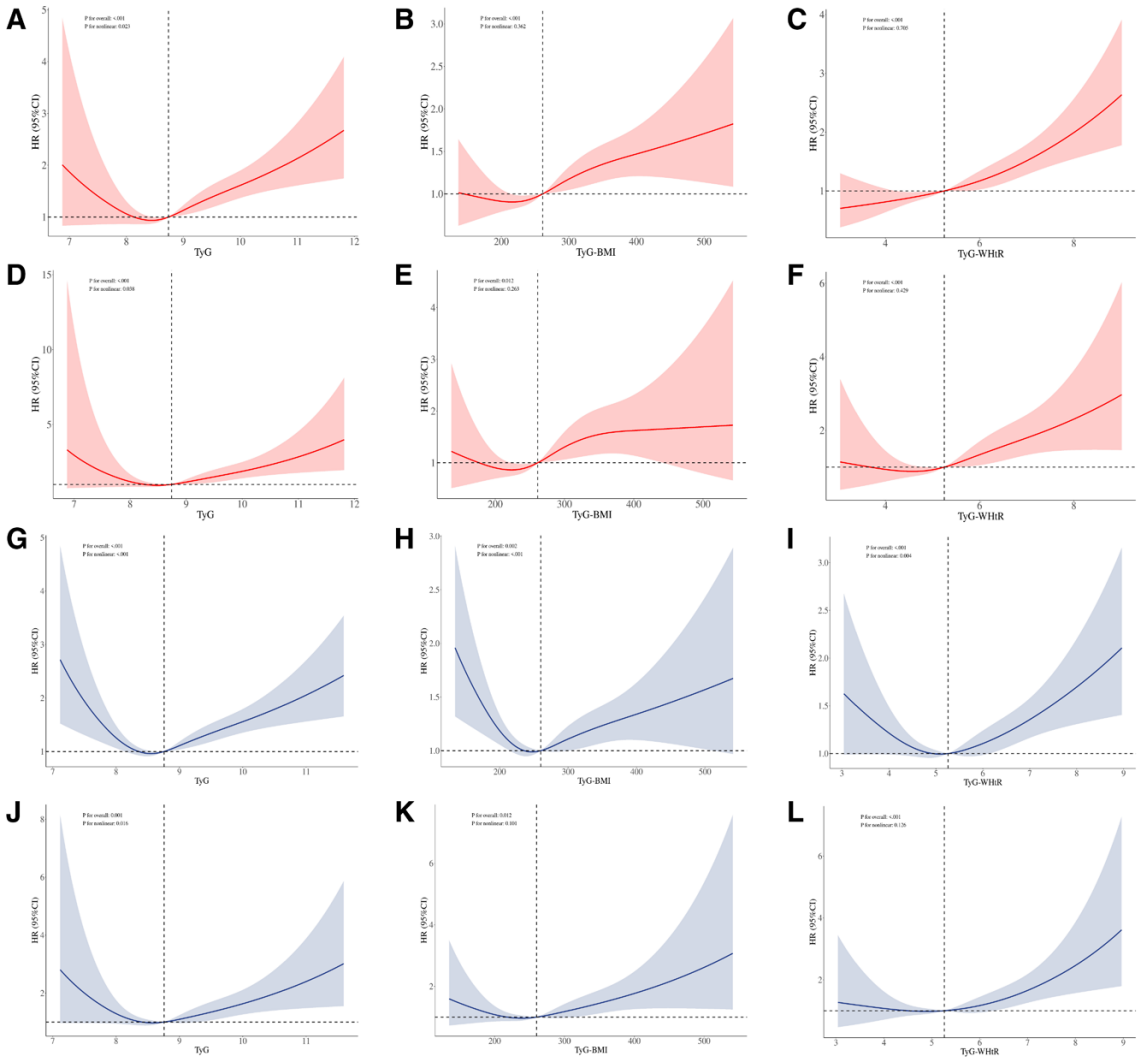
Restricted cubic spline analyses of TyG-derived indices in relation to all-cause and cardiovascular mortality risks in KDM-BA and PA populations. KDM-BA population: (A, D) TyG versus all-cause and cardiovascular mortality; (B, E) TyG-BMI index versus all-cause and cardiovascular mortality; (C, F) TyG-WHtR index versus all-cause and cardiovascular mortality. PA population: (G, J) TyG versus all-cause and cardiovascular mortality; (H, K) TyG-BMI index versus all-cause and cardiovascular mortality; and (I, L) TyG-WHtR index versus all-cause and cardiovascular mortality. Models III, adjusted for age, liver disease, cancer, gender, race, education, marital status, PIR, smoking status, drinking, CVD, CKD, ALT, AST, BUN, and UA. ALT = alanine aminotransferase, AST = aspartate aminotransferase, BUN = blood urea nitrogen, CI = confidence interval, CKD = chronic kidney disease, CVD = cardiovascular diseases, HR = hazard ratio, KDM-BA = Klemera–Doubal biological age acceleration, PA = phenotypic age acceleration, PIR = poverty-income ratio, SD = standard deviation, TyG = triglyceride-glucose index, TyG-BMI = TyG × body mass index, TyG-WHtR = TyG combined with waist-to-height ratio, UA = uric acid.

Using restricted cubic spline, we evaluated the nonlinear relationships between TyG and its related indices (Fig. 5A–F). In the KDM population, TyG exhibited significant nonlinear associations with all-cause mortality (nonlinear *P* = .023) and cardiovascular mortality (nonlinear *P* = .038). Threshold analysis revealed that the risk of all-cause mortality significantly increased when TyG exceeded 8.605, whereas the risk of cardiovascular mortality increased significantly when TyG surpassed 8.263 (Table S6, Supplemental Digital Content, https://links.lww.com/MD/Q724). No significant nonlinear relationships were observed between TyG-BMI and TyG-WHtR.

In the PA population, TyG demonstrated significant nonlinear associations with both all-cause mortality (nonlinear *P* = .016) and cardiovascular mortality (nonlinear *P* < .001), showing a clear dose-response relationship (Fig. 5G–L). Threshold analysis indicated that the risk of all-cause or cardiovascular mortality significantly increased when TyG exceeded 8.642 and 8.098, respectively. For TyG-BMI, the nonlinear relationship with all-cause mortality was highly significant (nonlinear *P* < .001), with a marked increase in mortality risk when TyG-BMI exceeded 215.145 (HR = 1.00, *P* = .031). TyG-WHtR also showed a significant nonlinear relationship with all-cause mortality (*P* for nonlinearity = .004), with the risk of all-cause mortality significantly increasing beyond a threshold of 5.034 (HR = 1.20, *P* < .001; Tables S7–S8, Supplemental Digital Content, https://links.lww.com/MD/Q724).

### 4.5. Subgroup analysis of the association between TyG-related indices and mortality

Subgroup analysis showed that age and severity of BA influenced the association between TyG-related indices and mortality. Specifically, the association between TyG-related indices and mortality risk was stronger in individuals aged < 65 years, whereas the association was somewhat weaker in those aged ≥ 65 years. Furthermore, individuals with more severe BA (e.g., KDM-BA and PA ≥ 5) showed a stronger association between TyG-related indices and mortality risk.

Although antihypertensive medication, statin, and antihyperglycemic agent use did not show a significant impact on the relationship between TyG-related indices and mortality, in some subgroups (e.g., statin use), the association between TyG-related indices and mortality showed a slight increase (HR: 1.30, 95% CI: 1.07–1.57, *P* = .007, *P* for interaction = .045). However, the influence of drug use on this relationship was limited (Tables S9–S10, Supplemental Digital Content, https://links.lww.com/MD/Q724).

## 5. Discussions

This study demonstrated that TyG and its related indices, TyG-BMI and TyG-WHtR, are significant predictors of all-cause and cardiovascular mortality in BA populations. These results highlight that higher levels of TyG and its derivatives were consistently associated with worse survival outcomes across different BA groups, particularly in the KDM-BA population. Notably, the associations between TyG-related indices and mortality risk were more pronounced in individuals under the age of 65 years and those exhibiting higher BA, further supporting the idea that metabolic dysregulation, as reflected by TyG, may play a crucial role in the aging process and its associated mortality risks. These findings underscore the potential utility of TyG and its derivatives as biomarkers for the early identification of individuals at a higher risk of mortality in aging populations.

This study revealed the prognostic value of TyG and its derived indices in BA subpopulations. The KDM-BA approach quantifies the molecular cumulative damage of BA by integrating multiple biological markers, such as inflammatory factors,^[24]^ metabolic enzymes,^[25,26]^ and epigenetic clocks^[27,28]^ whereas the PA approach reflects organ functional decline based on clinical phenotype parameters, such as albumin and ALP levels.^[22,29]^ As a key surrogate marker for IR, the TyG may accelerate the BA process through the metabolic aging-related mortality risk, involving 2 core pathways. First, mitochondrial-telomere axis damage: in IR states, the efficiency of oxidative phosphorylation in skeletal muscle and liver cells is reduced, leading to excessive production of reactive oxygen species (ROS). ROS activates the p53/p21 pathway, inducing cell cycle arrest and telomere attrition, a process that aligns with the acceleration of epigenetic age assessed by KDM-BA (e.g., DNA methylation clock shift).^[30,31]^ Notably, this ROS accumulation is amplified by impaired Nrf2-mediated antioxidant defenses: TyG elevation suppresses Nrf2 nuclear translocation by enhancing its binding to Keap1 (a cytoplasmic repressor), thereby reducing transcription of downstream antioxidants like HO-1 and NQO1. This blunted antioxidant response – consistent with Nrf2’s role as a “master regulator of redox homeostasis”exacerbates mitochondrial DNA damage and telomere shortening, particularly in KDM-BA populations sensitive to molecular perturbations.^[13]^ Survival analysis results indicate that in the KDM-BA population, high TyG levels are significantly associated with both all-cause and cardiovascular mortality (*P* < .001), suggesting that elevated TyG may accelerate cellular aging through mitochondrial damage and telomere attrition, thereby increasing the risk of mortality, particularly in individuals with metabolic disorders. Second, inflammation-endothelial dysfunction axis dysregulation: elevated TyG levels promote M1 polarization of adipose tissue macrophages, release pro-inflammatory cytokines (such as IL-6 and TNF-α), and induce the senescence-associated secretory phenotype^[32]^ which disrupts endothelial function and accelerates atherosclerotic plaque formation.^[33]^ This process is exacerbated by suppressed AMPK activity: IR inhibits AMPK phosphorylation at Thr172, impairing its ability to restrain NF-κB nuclear translocation and pro-inflammatory gene expression In PA populations, where organ functional decline predominates, TyG-related inflammation still drives cardiovascular mortality, as evidenced by the association between TyG and cardiovascular death – likely via amplified endothelial dysfunction and plaque instability, similar to how inflammation promotes postinfarction remodeling.^[12,15]^

This study further revealed the differential prognostic value of TyG-BMI and TyG-WHtR in the BA subpopulations. TyG-derived indices, by integrating IR with body composition parameters (such as obesity levels and visceral fat distribution), provide a more sensitive reflection of the cumulative effects of metabolic aging. The underlying mechanism may involve an imbalance in the adiponectin/leptin ratio caused by visceral fat accumulation, which exacerbates insulin signal transduction disorders by inhibiting AMPK activity and activating mTOR pathway.^[34–36]^ This metabolic-inflammation interaction aligns closely with the multi-omics characteristics of the KDM-BA assessment system, such as inflammatory markers^[37]^ and metabolic enzyme profiles.^[38]^ TyG-derived indices exhibited stronger predictive power for mortality risk in the KDM-BA population (TyG-BMI: HR range = 1.19–1.23, *P* < .05; TyG-WHtR: HR range = 1.28–1.34, *P* < .001). In contrast, PA assessment, which focuses on organ decline markers such as liver and kidney function and nutritional status, is less sensitive to metabolic abnormalities, leading to the lack of a significant association between TyG-BMI and mortality in the PA population. Only TyG-WHtR retained its independent predictive value for cardiovascular mortality (HR = 1.26, 95% CI 1.03–1.51). This heterogeneity suggests that visceral fat distribution (TyG-WHtR) may serve as a central hub linking metabolic disorders to organ function decline. Even in PA populations, TyG-WHtR may drive cardiovascular events through its pro-inflammatory microenvironment and hemodynamic load, potentially via AMPK/Nrf2 crosstalk: visceral fat-derived free fatty acids suppress AMPK, which normally phosphorylates and stabilizes Nrf2, creating a feedforward loop of oxidative stress and organ damage.^[39,40]^

The threshold effects revealed by the nonlinear analysis (TyG-BMI > 215.145, TyG-WHtR > 5.034) had significant clinical implications. When TyG-BMI exceeds this threshold, adipose tissue transitions from a compensatory metabolic phase (where lipids are safely stored in subcutaneous fat) to a decompensated phase (where ectopic fat is deposited in the liver and heart), leading to systemic damage mediated by free fatty acid spillover and lipotoxicity.^[41,42]^ At this tipping point, AMPK activity is severely blunted (as AMP/ATP ratio homeostasis is disrupted), impairing its regulation of fatty acid oxidation and mitochondrial biogenesis.simultaneously, Nrf2 is sequestered in the cytoplasm by Keap1, losing its ability to induce antioxidant genes.^[13,15]^ The TyG-BMI threshold (215.145) identified in this study aligns with the pathological tipping point of metabolic obesity,^[43]^ suggesting that the metabolic buffering capacity of visceral fat becomes saturated, significantly increasing mortality risk (HR = 1.48, 95% CI 1.22–1.79). TyG-WHtR, which integrates the waist-to-height ratio, more directly reflects the abnormal visceral fat distribution. Even in the PA population, where organ function decline is the primary characteristic, TyG-WHtR independently predicted cardiovascular mortality (HR = 1.26, 95% CI 1.03–1.51). The threshold effect of TyG-WHtR (>5.034) further underscores the central role of abdominal obesity: WHtR is more sensitive than BMI in reflecting visceral fat load,^[44,45]^ thus allowing the identification of high-risk individuals even within the PA population.

Subgroup analysis revealed that individuals under 65 years of age exhibited a stronger association between TyG-related indices and mortality risk, possibly because of the more pronounced long-term cumulative effects of metabolic disorders in younger populations. For example, in patients with metabolic dysfunction-associated fatty liver disease, the predictive sensitivity of the TyG-WHtR index for mortality is more prominent in the younger subgroup.^[46]^ This difference may relate to preserved AMPK/Nrf2 responsiveness in younger individuals: aging reduces AMPK activation capacity (via increased ST loop phosphorylation) and Nrf2 transcriptional efficiency (via Neh7 domain-mediated repression by retinoid X receptor alpha), blunting the metabolic-aging interaction in older populations.^[13,14]^ Older individuals also often present with comorbidities and compensatory physiological changes, which may partially offset the prognostic power of TyG for mortality risk.^[47,48]^

Importantly, in populations with higher levels of BA (e.g., KDM-BA ≥ 5 years), each 1-SD increase in the TyG was associated with a 32% increase in all-cause mortality risk (HR = 1.32, 95% CI 1.17–1.47) and a 43% increase in cardiovascular mortality risk (HR = 1.43, 95% CI 1.17–1.75). This gradient effect suggests that metabolic disorders (such as IR) and aging-related molecular damage (such as telomere attrition and protein homeostasis imbalance) may synergistically amplify mortality risk. The potential mechanism is that IR accelerates molecular damage through oxidative stress, whereas the decline in cellular repair capacity associated with aging exacerbates metabolic dysregulation, creating a vicious cycle.^[49,50]^

Additionally, the use of antihypertensive drugs, statins, and antidiabetic medications did not significantly alter the relationship between TyG levels and mortality in most cases. Although statin use showed a slight increase in the association, the effect was small, indicating that the impact of medication on the relationship between TyG and mortality was limited. This further supports the inherent independent association between metabolic indicators (such as TyG) and mortality risk, which may not be significantly influenced by pharmacological treatments for hypertension, diabetes, or dyslipidemia.Future studies should explore whether AMPK activators (e.g., 5-aminoimidazole-4-carboxamide ribonucleotide) or Nrf2 inducers (e.g., sulforaphane) can mitigate the TyG-related mortality risk, particularly in high-risk subgroups such as KDM-BA ≥ 5 years.

## 6. Strengths and limitations

The strengths of this study lie in utilizing a large, nationally representative sample to minimize sampling bias, conducting rigorous statistical adjustment for potential confounding factors (e.g., demographic characteristics and underlying diseases) to enhance the reliability of the results, and performing a systematic analysis of multiple TyG-derived indices – within the framework of 2 fully validated BA assessment methods – which enriches the dimensions of evaluation.

However, the study still has several limitations. First, as an observational study, it can only identify statistical associations of the TyG and its related indices with mortality, but cannot establish a causal relationship. Future validation via randomized controlled trials and investigation into causal mechanisms are therefore needed. Second, although adjustments have been made for key confounding factors, unmeasured variables (e.g., genetic susceptibility and long-term environmental exposures) may introduce residual confounding, which could restrict the generalizability of the findings. Third, lifestyle factors such as smoking and alcohol consumption were assessed via self-report, a method susceptible to recall bias or social desirability bias. Fourth, the BA model employs a cross-sectional approach, which fails to dynamically capture the aging process; incorporating longitudinal data would better enhance the accuracy of association assessments. Finally, while adjustments have been made for common medications, critical information (e.g., drug dosage, duration of use, and medication adherence) was unavailable, potentially compromising the accuracy of association estimates.

## 7. The clinical implications

The clinical implications of our findings are as follows: First, it provides a convenient and subtype-adapted tool for stratifying mortality risk in biologically aging populations. The TyG and its related indices (TyG-BMI and TyG-WHtR) can be calculated solely based on routine physical examination data and can be fully applied in biologically aging populations.

Second, it clarifies quantifiable metabolic intervention thresholds: the TyG has a key threshold of 8.1 to 8.6 (mortality risk increases significantly when exceeding this range), and in the PA population, mortality risk surges when TyG-BMI > 215.145 or TyG-WHtR > 5.034, which provides specific basis for formulating personalized metabolic control targets in clinical practice. Third, it supports personalized management based on BA characteristics: for populations with high aging burden (KDM-BA/PA ≥ 5), the follow-up interval for TyG should be shortened and stricter control targets established; meanwhile, combined with the subgroup analysis result that medications have limited impact on the association between TyG and mortality, lifestyle interventions (e.g., low-GI diet, aerobic exercise) are prioritized as the core intervention when thresholds are exceeded, facilitating precise metabolic health management in aging populations.

## 8. Conclusions

This study underscores the significance of TyG and its related indices (TyG-BMI and TyG-WHtR) as key indicators of all-cause and cardiovascular mortality in biologically aging populations. Notably, strong associations were found in the KDM-BA cohort, particularly among individuals aged < 65 years and those with advanced BA. These findings suggest that TyG and its related indices may play a crucial role in the long-term management of biologically aging populations and serve as valuable biomarkers for identifying high-risk individuals. This further highlights the importance of metabolic dysregulation in the aging process and its contribution to an increased mortality risk.

## Author contributions

**Conceptualization:** Meng-Qun Cheng, Zhong-Ping Bai, Gao Song, Cai-Qiong Zhang, Xing Liu, Rong Li.

**Data curation:** Meng-Qun Cheng, Gao Song, Xing Liu, Rong Li.

**Formal analysis:** Meng-Qun Cheng, Zhong-Ping Bai, Gao Song, Cai-Qiong Zhang, Xing Liu, Rong Li.

**Funding acquisition:** Gao Song, Rong Li.

**Investigation:** Gao Song, Cai-Qiong Zhang, Xing Liu, Rong Li.

**Methodology:** Meng-Qun Cheng, Zhong-Ping Bai, Gao Song, Cai-Qiong Zhang, Rong Li.

**Project administration:** Meng-Qun Cheng, Zhong-Ping Bai, Gao Song, Rong Li.

**Resources:** Zhong-Ping Bai, Gao Song, Rong Li.

**Software:** Zhong-Ping Bai, Gao Song, Rong Li.

**Supervision:** Meng-Qun Cheng, Zhong-Ping Bai, Gao Song, Rong Li.

**Validation:** Meng-Qun Cheng, Zhong-Ping Bai, Gao Song, Cai-Qiong Zhang, Xing Liu, Rong Li.

**Visualization:** Zhong-Ping Bai, Gao Song, Cai-Qiong Zhang, Rong Li.

**Writing – original draft:** Meng-Qun Cheng, Zhong-Ping Bai, Gao Song, Cai-Qiong Zhang, Rong Li.

**Writing – review & editing:** Gao Song, Rong Li.

## Supplementary Material


