# Supplementary material for: The association of triglyceride-glucose index and related indices with all-cause and cardiovascular mortality in biologically aging populations
Source: Medicine (Baltimore). 2025 Nov 21;104(47):e46036. doi: 10.1097/MD.0000000000046036 (PMC12643700; doi:10.1097/MD.0000000000046036)
Supplement: Supplementary file 1 [file medi-104-e46036-s001.pdf]

Figure S1 Continuous Variable Distribution Analysis in KDM-BA populations

Abbreviations: ALT, alanine aminotransferase; AST, aspartate aminotransferase; BUN, blood urea nitrogen; KDM-BA, Klemera–Doubal biological age acceleration; PA, phenotypic age acceleration; TG, triglyceride; TyG, triglyceride-glucose index; TyG-BMI, triglyceride-glucose index combined with BMI; TyG-WHtR, triglyceride-glucose index combined with waist-to-height ratio; CVD, cardiovascular disease; CKD, chronic kidney disease; FBG, fasting blood glucose; BMI, body mass index.

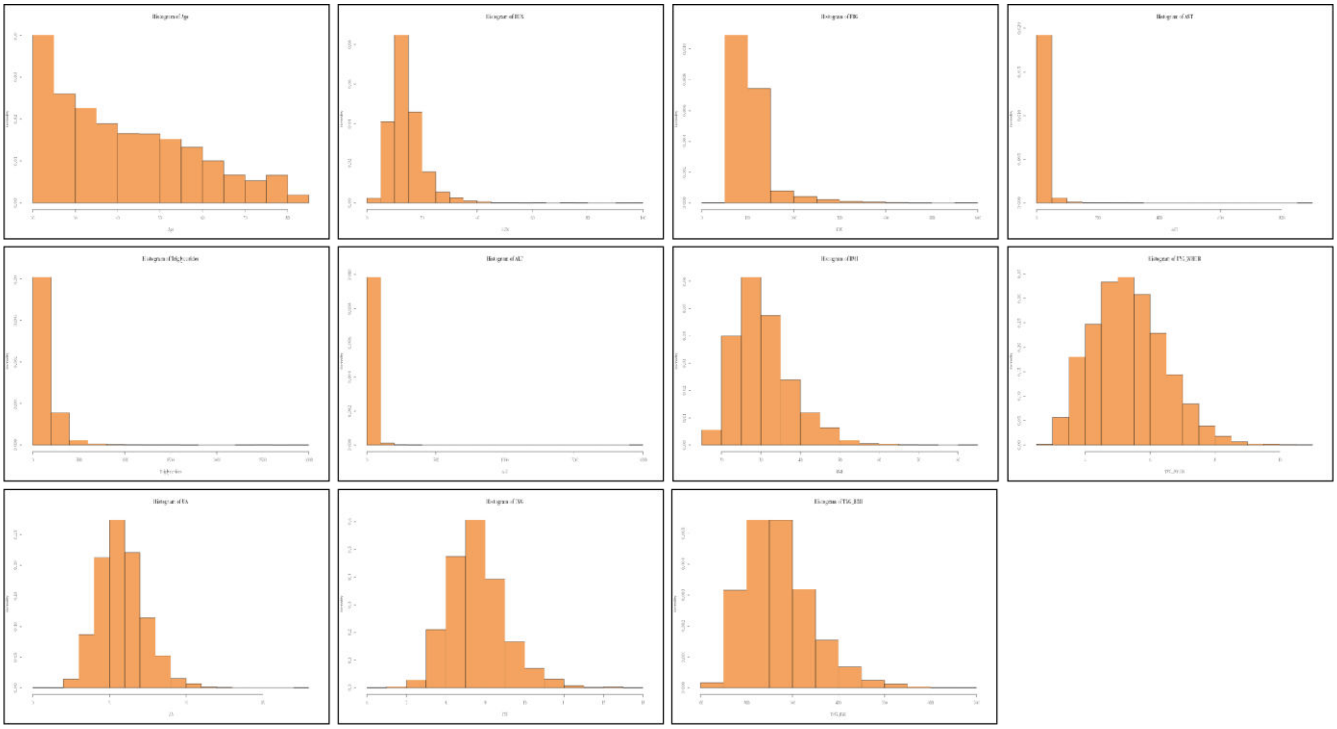

Figure S2 Continuous Variable Distribution Analysis in PA populations

Abbreviations: ALT, alanine aminotransferase; AST, aspartate aminotransferase; BUN, blood urea nitrogen; KDM-BA, Klemera–Doubal biological age acceleration; PA, phenotypic age acceleration; TG, triglyceride; TyG, triglyceride-glucose index; TyG-BMI, triglyceride-glucose index combined with BMI; TyG-WHtR, triglyceride-glucose index combined with waist-to-height ratio; CVD, cardiovascular disease; CKD, chronic kidney disease; FBG, fasting blood glucose; BMI, body mass index.

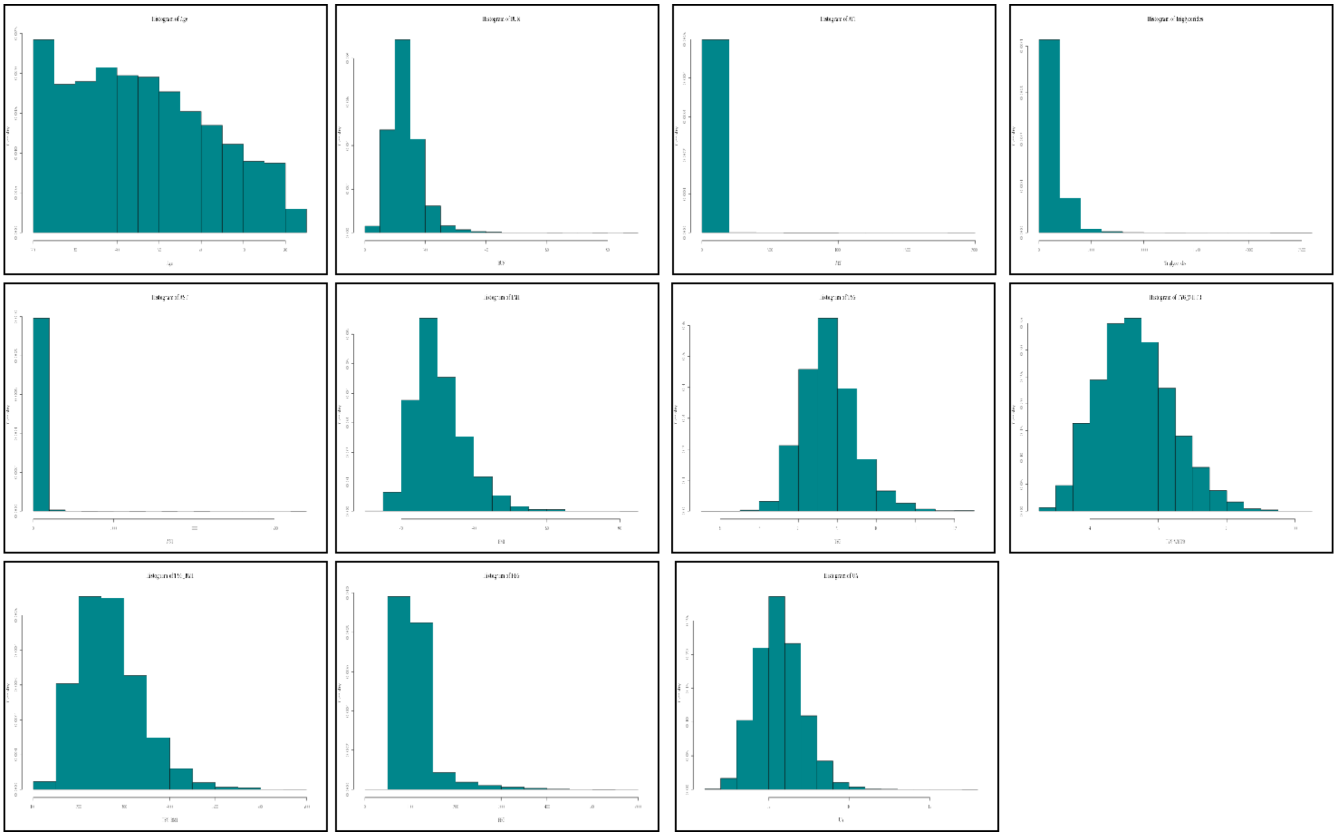

Table S1. Missing Data Overview of Variables in KDM-BA and PA Populations

Variables	KDM-BA population(n = 7340)		PA population(n = 7839)	
	Freq	Miss (Percent)	Freq	Miss (Percent)
Age	7,340	0 (0.00)	7,839	0 (0.00)
ALT(U/L)	7,335	**5 (0.07)**	7,831	**8 (0.10)**
AST(U/L)	7,330	**10 (0.14)**	7,828	**11 (0.14)**
BUN, mg/dL	7,340	0 (0.00)	7,839	0 (0.00)
TG, mg/dL	7,336	**4 (0.05)**	7,838	**1 (0.01)**
Uric acid, mg/dL	7,340	0 (0.00)	7,839	0 (0.00)
FBG, mg/dL	7,340	0 (0.00)	7,839	0 (0.00)
BMI, kg/m²	7,340	0 (0.00)	7,839	0 (0.00)
Antihypertensive use, %	2,995	**4345 (59.20)**	2,976	**4863 (62.04)**
Statin use, %	3,378	**3962 (53.98)**	3,413	**4426 (56.46)**
Antihyperglycemic agent use, %	1,560	**5780 (78.75)**	1,729	**6110 (77.94)**
Liver disease, %	7,340	0 (0.00)	7,839	0 (0.00)
Cancer, %	7,340	0 (0.00)	7,839	0 (0.00)
Gender, %	7,340	0 (0.00)	7,839	0 (0.00)
Race, %	7,340	0 (0.00)	7,839	0 (0.00)
Education, %	7,330	**10 (0.14)**	7,829	**10 (0.13)**
Maritalstatus, %	7,269	**71 (0.97)**	7,729	**110 (1.40)**
Poverty income ratio	6,724	**616 (8.39)**	7,120	**719 (9.17)**
Smoking status, %	7,332	**8 (0.11)**	7,832	**7 (0.09)**
Drinking, %	6,065	**1275 (17.37)**	6,246	**1593 (20.32)**
TyG	7,340	0 (0.00)	7,839	0 (0.00)
TyG-BMI	7,340	0 (0.00)	7,839	0 (0.00)
TyG-WHtR	7,340	0 (0.00)	7,839	0 (0.00)
All-cause mortality, %	7,340	0 (0.00)	7,839	0 (0.00)
Cardiovascular mortality, %	7,340	0 (0.00)	7,839	0 (0.00)
CVD, %	7,266	**74 (1.01)**	7,748	**91 (1.16)**
CKD, %	7,303	**37 (0.50)**	7,839	0 (0.00)

Abbreviations: ALT, alanine aminotransferase; AST, aspartate aminotransferase; BUN, blood urea nitrogen; KDM-BA, Klemera–Doubal biological age acceleration; PA, phenotypic age acceleration; TG, triglyceride; TyG, triglyceride-glucose index; TyG-BMI, triglyceride-glucose index combined with BMI; TyG-WHtR, triglyceride-glucose index combined with waist-to-height ratio; CVD, cardiovascular disease; CKD, chronic kidney disease; FBG, fasting blood glucose; BMI, body mass index.

Table S2 Covariance analysis of TyG-related Indices and other variables

	KDM-BA population			PA population		
	GVIF	Df	$GVIF^{1/2Df}$	GVIF	Df	$GVIF^{1/2Df}$
**TyG index**						
Age	1.952	1	1.397	1.655	1	1.287
ALT	1.38	1	1.175	2.252	1	1.501
AST	1.36	1	1.166	2.231	1	1.494
BUN	1.69	1	1.3	1.498	1	1.224
Cancer	1.108	1	1.053	1.124	1	1.06
CKD	1.493	1	1.222	1.299	1	1.14
CVD	1.263	1	1.124	1.197	1	1.094
Drinking	1.196	1	1.094	1.198	1	1.094
Education	1.346	2	1.077	1.332	2	1.074
Gender	1.306	1	1.143	1.338	1	1.157
Liver disease	1.026	1	1.013	1.024	1	1.012
Marital status	1.088	1	1.043	1.093	1	1.045
PIR	1.248	2	1.057	1.304	2	1.069
Race	1.358	3	1.052	1.36	3	1.053
Smoking status	1.351	2	1.078	1.327	2	1.073
TyG	1.219	1	1.104	1.158	1	1.076
UA	1.366	1	1.169	1.347	1	1.16
**TyG-BMI index**						
Age	1.879	1	1.371	1.64	1	1.28
ALT	1.376	1	1.173	2.245	1	1.498
AST	1.362	1	1.167	2.228	1	1.493
BUN	1.693	1	1.301	1.504	1	1.226

Cancer	1.108	1	1.053	1.123	1	1.06
CKD	1.479	1	1.216	1.277	1	1.13
CVD	1.266	1	1.125	1.197	1	1.094
Drinking	1.201	1	1.096	1.201	1	1.096
Education	1.344	2	1.077	1.327	2	1.073
Gender	1.32	1	1.149	1.402	1	1.184
Liver disease	1.026	1	1.013	1.024	1	1.012
Maritalstatus	1.093	1	1.045	1.097	1	1.048
PIR	1.25	2	1.057	1.304	2	1.069
Race	1.302	3	1.045	1.302	3	1.045
Smoking status	1.351	2	1.078	1.339	2	1.076
TyG-BMI	1.154	1	1.074	1.162	1	1.078
UA	1.468	1	1.212	1.446	1	1.203
**TyG-WHtR index**						
Age	1.929	1	1.389	1.664	1	1.29
ALT	1.378	1	1.174	2.248	1	1.499
AST	1.362	1	1.167	2.232	1	1.494
BUN	1.706	1	1.306	1.509	1	1.228
Cancer	1.108	1	1.052	1.123	1	1.06
CKD	1.486	1	1.219	1.284	1	1.133
CVD	1.267	1	1.126	1.199	1	1.095
Drinking	1.203	1	1.097	1.202	1	1.097
Education	1.346	2	1.077	1.33	2	1.074
Gender	1.339	1	1.157	1.421	1	1.192
Liver disease	1.026	1	1.013	1.025	1	1.012
Marital status	1.094	1	1.046	1.097	1	1.047

PIR	1.251	2	1.058	1.304	2	1.069
Race	1.314	3	1.047	1.329	3	1.049
Smokingstatus	1.351	2	1.078	1.337	2	1.075
TyG-WHtR	1.264	1	1.124	1.249	1	1.118
UA	1.457	1	1.207	1.429	1	1.196

Abbreviations: ALT, alanine aminotransferase; AST, aspartate aminotransferase; BUN, blood urea nitrogen; KDM-BA, Klemera–Doubal biological age acceleration; PA, phenotypic age acceleration; TG, triglyceride; TyG, triglyceride-glucose index; TyG-BMI, triglyceride-glucose index combined with BMI; TyG-WHtR, triglyceride-glucose index combined with waist-to-height ratio; CVD, cardiovascular disease; CKD, chronic kidney disease; FBG, fasting blood glucose; BMI, body mass index.

Table S3 Cox Regression Analysis of TyG Index and Mortality in KDM-BA and PA Populations

Subgroup	KDM-BA population						PA population					
	Model 1		Model 2		Model 3		Model 1		Model 2		Model 3	
	HR (95% CI)	*P*	HR (95% CI)	*P*	HR (95% CI)	*P*	HR (95% CI)	*P*	HR (95% CI)	*P*	HR (95% CI)	*P*
**All-cause mortality**												
TyG(Per 1 SD)	1.59 (1.49 - 1.70)	**<.001**	1.29 (1.18 - 1.42)	**<.001**	1.23 (1.11 - 1.36)	<.001	1.42 (1.35 - 1.50)	**<.001**	1.20 (1.13 - 1.28)	**<.001**	1.16 (1.08 - 1.24)	**<.001**
Categories												
Q1	1.00 (Reference)		1.00 (Reference)		1.00 (Reference)		1.00 (Reference)		1.00 (Reference)		1.00 (Reference)	
Q2	1.30 (1.02 - 1.66)	**0.032**	0.78 (0.61 - 0.99)	**0.04**	0.79 (0.58 - 1.09)	0.147	1.34 (1.03 - 1.75)	**0.032**	0.84 (0.66 - 1.06)	0.142	0.88 (0.64 - 1.20)	0.421
Q3	2.15 (1.65 - 2.82)	**<.001**	1.07 (0.83 - 1.37)	0.621	1.06 (0.76 - 1.47)	0.732	1.62 (1.28 - 2.05)	**<.001**	0.94 (0.77 - 1.14)	0.535	0.94 (0.71 - 1.24)	0.659
Q4	3.60 (2.81 - 4.61)	**<.001**	1.48 (1.15 - 1.91)	**0.002**	1.36 (0.97 - 1.92)	0.077	2.64 (2.14 - 3.27)	**<.001**	1.29 (1.05 - 1.58)	**0.014**	1.27 (0.97 - 1.66)	0.078
P for trend		**<.001**		**<.001**		0.003		**<.001**		**<.001**		**0.005**
**Cardiovascular mortality**												
TyG(Per 1 SD)	1.68 (1.48 - 1.91)	**<.001**	1.39 (1.17 - 1.66)	**<.001**	1.30 (1.08 - 1.56)	0.005	1.46 (1.31 - 1.62)	**<.001**	1.25 (1.08 - 1.45)	**0.003**	1.19 (1.02 - 1.37)	**0.025**
Categories												
Q1	1.00 (Reference)		1.00 (Reference)		1.00 (Reference)		1.00 (Reference)		1.00 (Reference)		1.00 (Reference)	
Q2	1.49 (0.93 - 2.39)	0.096	0.86 (0.56 - 1.34)	0.509	0.95 (0.55 - 1.63)	0.845	1.70 (1.15 - 2.50)	**0.008**	1.01 (0.71 - 1.43)	0.968	1.07 (0.66 - 1.72)	0.795
Q3	2.17 (1.43 - 3.31)	**<.001**	1.08 (0.71 - 1.64)	0.727	1.09 (0.65 - 1.82)	0.75	1.72 (1.18 - 2.51)	**0.005**	0.97 (0.67 - 1.41)	0.894	1.07 (0.65 - 1.75)	0.792

Q4	4.41 (2.93 - 6.64)	**<.001**	1.82 (1.18 - 2.83)	**0.007**	1.60 (0.95 - 2.69)	0.077	3.10 (2.23 - 4.32)	**<.001**	1.51 (1.06 - 2.14)	**0.023**	1.48 (0.96 - 2.27)	0.076
P for trend		**<.001**		**<.001**		**0.024**		**<.001**		**0.013**		**0.029**

Abbreviations: HR, hazard ratio; CI, confidence interval; KDM-BA, Klemera–Doubal biological age acceleration; PA, phenotypic age acceleration; TyG, triglyceride-glucose index;

Model 1: Non-adjusted

Model 2: Adjusted for age, gender, and race

Model 3: Adjusted for Age, Liverdisease, Cancer, Gender, Race, Education, Marital status, PIR, Smoking status, Drinking, CVD, CKD, ALT, AST, BUN, UA.

Table S4 Cox Regression Analysis of TyG-BMI Index and Mortality in KDM-BA and PA Populations

Subgroup	KDM-BA population						PA population					
	Model 1		Model 2		Model 3		Model 1		Model 2		Model 3	
	HR (95% CI)	*P*	HR (95% CI)	*P*	HR (95% CI)	*P*	HR (95%CI)	*P*	HR (95% CI)	*P*	HR (95% CI)	*P*
All-cause mortality												
TyG-BMI(Per 1 SD)	1.19 (1.10 - 1.27)	<.001	1.15 (1.05 - 1.26)	0.003	1.19 (1.07 - 1.31)	0.001	1.05 (0.97 - 1.13)	0.214	1.00 (0.92 - 1.09)	0.996	1.03 (0.93 - 1.13)	0.609
Categories												
Q1	1.00 (Reference)		1.00 (Reference)		1.00 (Reference)		1.00 (Reference)		1.00 (Reference)		1.00 (Reference)	
Q2	1.36 (1.04 - 1.77)	**0.023**	0.91 (0.72 - 1.15)	0.417	1.00 (0.77 - 1.31)	0.973	0.99 (0.83 - 1.19)	0.943	0.80 (0.67 - 0.95)	**0.01**	0.87 (0.71 - 1.07)	0.178
Q3	1.30 (1.03 - 1.65)	**0.028**	0.95 (0.77 - 1.17)	0.625	0.98 (0.76 - 1.27)	0.886	1.05 (0.85 - 1.29)	0.655	0.80 (0.67 - 0.96)	**0.019**	0.86 (0.69 - 1.08)	0.187
Q4	1.66 (1.34 - 2.08)	**<.001**	1.24 (0.99 - 1.57)	0.064	1.48 (1.10 - 2.00)	**0.01**	1.11 (0.89 - 1.38)	0.36	0.94 (0.76 - 1.15)	0.524	1.04 (0.80 - 1.36)	0.755
P for trend		**<.001**		**0.038**		**0.006**		0.32		0.526		0.77
Cardiovascular mortality												
TyG-BMI(Per 1 SD)	1.30 (1.15 - 1.47)	<.001	1.35 (1.15 - 1.59)	<.001	1.23 (1.05 - 1.46)	0.013	1.19 (1.03 - 1.36)	**0.015**	1.23 (1.02 - 1.50)	**0.032**	1.18 (0.97 - 1.44)	0.089
Categories												
Q1	1.00 (Reference)		1.00 (Reference)		1.00 (Reference)		1.00 (Reference)		1.00 (Reference)		1.00 (Reference)	
Q2	1.85 (1.25 - 2.73)	**0.002**	1.22 (0.85 - 1.75)	0.273	1.35 (0.84 - 2.18)	0.214	1.29 (0.88 - 1.89)	0.187	0.99 (0.72 - 1.36)	0.945	0.98 (0.65 - 1.46)	0.905

Q3	1.59 (1.06 - 2.40)	**0.026**	1.24 (0.82 - 1.86)	0.308	1.09 (0.67 - 1.78)	0.739	1.22 (0.89 - 1.68)	0.218	0.93 (0.70 - 1.24)	0.625	0.96 (0.67 - 1.40)	0.848
Q4	2.47 (1.69 - 3.60)	**<.001**	2.05 (1.34 - 3.14)	**<.001**	1.98 (1.17 - 3.33)	0.011	1.63 (1.13 - 2.37)	**0.009**	1.50 (1.05 - 2.15)	**0.028**	1.47 (0.93 - 2.32)	0.097
P for trend		**<.001**		0.002		0.022		**0.016**		0.055		0.114

Abbreviations: HR, hazard ratio; CI, confidence interval; KDM-BA, Klemera–Doubal biological age acceleration; PA, phenotypic age acceleration; TyG, triglyceride-glucose index; TyG-BMI, TyG combined with BMI; BMI, body mass index.

Model 1: Non-adjusted

Model 2: Adjusted for age, gender, and race

Model 3: Adjusted for Age, Liverdisease, Cancer, Gender, Race, Education, Marital status, PIR, Smoking status, Drinking, CVD, CKD, ALT, AST, BUN, UA.

Table S5 Cox Regression Analysis of TyG-WHtR Index and Mortality in KDM-BA and PA Populations

Subgroup	KDM-BA population						PA population					
	Model 1		Model 2		Model 3		Model 1		Model 2		Model 3	
	HR (95% CI)	*P*	HR (95% CI)	*P*	HR (95% CI)	*P*	HR (95% CI)	*P*	HR (95% CI)	*P*	HR (95% CI)	*P*
All-cause mortality												
TyG-WHtR(Per 1 SD)	1.56 (1.46 - 1.66)	<.001	1.28 (1.16 - 1.41)	<.001	1.28 (1.15 - 1.44)	<.001	1.39 (1.30 - 1.49)	**<.001**	1.12 (1.03 - 1.21)	**0.011**	1.10 (1.00 - 1.21)	0.055
Categories												
Q1	1.00 (Reference)		1.00 (Reference)		1.00 (Reference)		1.00 (Reference)		1.00 (Reference)		1.00 (Reference)	
Q2	2.09 (1.57 - 2.78)	**<.001**	1.03 (0.80 - 1.31)	0.839	1.18 (0.81 - 1.72)	0.378	1.59 (1.26 - 2.00)	**<.001**	0.93 (0.76 - 1.15)	0.522	1.00 (0.78 - 1.26)	0.969
Q3	2.61 (1.90 - 3.59)	**<.001**	1.07 (0.80 - 1.43)	0.652	1.24 (0.83 - 1.86)	0.301	1.82 (1.40 - 2.35)	**<.001**	0.80 (0.64 - 0.99)	**0.041**	0.86 (0.67 - 1.10)	0.23
Q4	3.83 (2.99 - 4.91)	**<.001**	1.60 (1.23 - 2.09)	**<.001**	1.79 (1.20 - 2.68)	0.004	2.49 (1.98 - 3.14)	**<.001**	1.18 (0.95 - 1.48)	0.14	1.19 (0.91 - 1.58)	0.207
P for trend		<.001		**<.001**		<.001		**<.001**		0.133		0.221
Cardiovascular mortality												
TyG-WHtR(Per 1 SD)	1.56 (1.46 - 1.66)	<.001	1.50 (1.28 - 1.76)	<.001	1.34 (1.13 - 1.58)	<.001	1.56 (1.38 - 1.77)	**<.001**	1.35 (1.11 - 1.65)	**0.002**	1.26 (1.03 - 1.53)	**0.022**
Categories												
Q1	1.00 (Reference)		1.00 (Reference)		1.00 (Reference)		1.00 (Reference)		1.00 (Reference)		1.00 (Reference)	
Q2	1.93 (1.11 - 3.35)	**0.02**	0.87 (0.51 - 1.48)	0.599	0.91 (0.49 - 1.68)	0.762	1.29 (0.88 - 1.89)	0.187	0.99 (0.72 - 1.36)	0.945	0.98 (0.65 - 1.46)	0.905

Q3	2.76 (1.51 - 5.05)	**<.001**	1.09 (0.63 - 1.89)	0.764	1.06 (0.59 - 1.90)	0.852	1.22 (0.89 - 1.68)	0.218	0.93 (0.70 - 1.24)	0.625	0.96 (0.67 - 1.40)	0.848
Q4	4.56 (2.69 - 7.73)	**<.001**	1.91 (1.11 - 3.29)	**0.019**	1.62 (0.84 - 3.13)	**0.146**	1.63 (1.13 - 2.37)	**0.009**	1.50 (1.05 - 2.15)	**0.028**	1.47 (0.93 - 2.32)	0.097
P for trend		<.001		**<.001**		0.016		**<.001**		**0.01**		0.087

Abbreviations: HR, hazard ratio; CI, confidence interval; KDM-BA, Klemera–Doubal biological age acceleration; PA, phenotypic age acceleration; TyG, triglyceride-glucose index; TyG-WHTR, TyG combined with waist-to-height ratio.

Model 1: Non-adjusted

Model 2: Adjusted for age, gender, and race

Model 3: Adjusted for Age, Liverdisease, Cancer, Gender, Race, Education, Marital status, PIR, Smoking status, Drinking, CVD, CKD, ALT, AST, BUN, UA.

Table S6 Threshold Effect Analysis of TyG Index on Mortality in KDM-BA and PA Populations

	KDM-BA population			PA population	
	Adjusted HR (95% CI)	*P*		Adjusted HR (95% CI)	*P*
**All-cause mortality**					
Model 1 Fitting model by standard linear regression	1.23 (1.12 - 1.35)	**<.001**		1.14 (1.05 - 1.25)	**0.003**
Model 2 Fitting model by two-piecewise linear regression					
Inflection point					
<8.605	0.70 (0.44 - 1.10)	0.121	<8.642	0.65 (0.44 - 0.95)	**0.025**
≥8.605	1.38 (1.22 - 1.55)	**<.001**	≥8.642	1.31 (1.16 - 1.48)	**<.001**
P for likelihood test		**0.013**			**<.001**
**Cardiovascular mortality**					
Model 1 Fitting model by standard linear regression	1.37 (1.18 - 1.60)	**<.001**		1.27 (1.09 - 1.48)	**0.002**
Model 2 Fitting model by two-piecewise linear regression					
Inflection point					
<8.263	0.15 (0.03 - 0.73)	**0.018**	<8.098	0.05 (0.01 - 0.27)	**<.001**
≥8.263	1.52 (1.28 - 1.79)	**<.001**	≥8.098	1.37 (1.16 - 1.61)	**<.001**
P for likelihood test		**0.045**			**0.041**

Abbreviations: HR, hazard ratio; CI, confidence interval; TyG, triglyceride-glucose index; KDM-BA, Klemera–Doubal biological age acceleration; PA, phenotypic age acceleration.Models were adjusted for age, Liverdisease, Cancer, Gender, Race, Education, Marital status, PIR, Smoking status, Drinking, CVD, CKD, ALT, AST, BUN, UA.

Table S7 Threshold Effect Analysis of TyG-BMI Index on Mortality in PA Populations

	PA population	
	Adjusted HR (95% CI)	*P*
**All-cause mortality**		
Model 1 Fitting model by standard linear regression	1.00 (1.00 - 1.00)	0.603
Model 2 Fitting model by two-piecewise linear regression		
Inflection point		
<215.145	0.99 (0.98 - 0.99)	<.001
≥215.145	1.00 (1.00 - 1.00)	0.031
P for likelihood test		<.001

Abbreviations: HR, hazard ratio; CI, confidence interval; TyG, triglyceride-glucose index; TyG-BMI, TyG combined with BMI; PA, phenotypic age acceleration. Models were adjusted for age, Liverdisease, Cancer, Gender, Race, Education, Marital status, PIR, Smoking status, Drinking, CVD, CKD, ALT, AST, BUN, UA.

Table S8 Threshold Effect Analysis of TyG-WHtR Index on Mortality in PA Populations

	PA population	
	Adjusted HR (95% CI)	*P*
**All-cause mortality**		
Model 1 Fitting model by standard linear regression	1.05 (0.98 - 1.12)	0.168
Model 2 Fitting model by two-piecewise linear regression		
Inflection point		
<5.034	0.65 (0.50 - 0.84)	0.001
≥5.034	1.20 (1.09 - 1.31)	<.001
P for likelihood test		<.001

Abbreviations: HR, hazard ratio; CI, confidence interval; TyG, triglyceride-glucose index; TyG-WHtR, TyG combined with waist-to-height ratio; PA, phenotypic age acceleration.Models were adjusted for age, Liverdisease, Cancer, Gender, Race, Education, Marital status, PIR, Smoking status, Drinking, CVD, CKD, ALT, AST, BUN, UA.

Table S9 Subgroup Analysis of Adjusted HRs for All-Cause Mortality Associated with TyG-related Indices in The KDM-BA Population

Variables	TyG			P for interaction	TyG-BMI			P for interaction	TyG-WHtR			P for interaction
	n (%)	HR (95% CI)	*P*		n (%)	HR (95% CI)	*P*		n (%)	HR (95% CI)	*P*	
**All-cause mortality**												
Age				**0.001**				0.155				**0.04**
＜65	5461 (74.40)	1.40 (1.22 ~ 1.60)	**<.001**		5461 (74.40)	1.32 (1.14 ~ 1.53)	**<.001**		5461 (74.40)	1.51 (1.27 ~ 1.79)	**<.001**	
≥65	1879 (25.60)	1.14 (1.01 ~ 1.30)	**0.033**		1879 (25.60)	1.17 (1.02 ~ 1.35)	**0.026**		1879 (25.60)	1.24 (1.09 ~ 1.42)	**0.001**	
Antihypertensive use, n%				0.514				0.779				0.489
Yes	2483 (82.90)	1.26 (1.11 ~ 1.43)	**<.001**		2483 (82.90)	1.14 (1.00 ~ 1.29)	**0.044**		2483 (82.90)	1.24 (1.08 ~ 1.42)	**0.003**	
No	512 (17.10)	1.57 (1.03 ~ 2.39)	**0.034**		512 (17.10)	1.65 (0.99 ~ 2.73)	0.054		512 (17.10)	1.80 (1.09 ~ 2.98)	**0.022**	
Statin use				**0.045**				0.5				0.698
Yes	1442 (42.69)	1.30 (1.07 ~ 1.57)	**0.007**		1442 (42.69)	1.19 (0.99 ~ 1.43)	0.071		1442 (42.69)	1.24 (1.02 ~ 1.51)	**0.03**	
No	1936 (57.31)	0.94 (0.76 ~ 1.17)	0.602		1936 (57.31)	1.08 (0.88 ~ 1.31)	0.459		1936 (57.31)	1.14 (0.91 ~ 1.42)	0.247	
Antihyperglycemic agent use, n%				0.504				0.401				0.677
Yes	859 (55.06)	1.27 (1.05 ~ 1.55)	**0.016**		859 (55.06)	1.09 (0.88 ~ 1.35)	0.419		859 (55.06)	1.18 (0.94 ~ 1.48)	0.163	
No	701 (44.94)	1.52 (1.19 ~ 1.94)	**<.001**		701 (44.94)	1.08 (0.85 ~ 1.37)	0.514		701 (44.94)	1.18 (0.91 ~ 1.54)	0.212	

KDM-BA				**0.001**				0.12				0.121
＜5	2570 (35.01)	0.98 (0.84 ~ 1.15)	0.825		2570 (35.01)	1.04 (0.84 ~ 1.29)	0.705		2570 (35.01)	1.07 (0.88 ~ 1.31)	0.508	
≥5	4770 (64.99)	1.32 (1.17 ~ 1.48)	**<.001**		4770 (64.99)	1.21 (1.08 ~ 1.35)	**<.001**		4770 (64.99)	1.33 (1.18 ~ 1.49)	**<.001**	
**Cardiovascular mortality**												
Age				0.556				0.89				0.86
＜65	5461 (74.40)	1.34 (1.03 ~ 1.74)	**0.029**		5461 (74.40)	1.32 (1.00 ~ 1.75)	0.051		5461 (74.40)	1.45 (1.06 ~ 1.99)	**0.019**	
≥65	1879 (25.60)	1.30 (1.05 ~ 1.61)	**0.015**		1879 (25.60)	1.28 (1.01 ~ 1.62)	**0.038**		1879 (25.60)	1.38 (1.09 ~ 1.76)	**0.008**	
Antihypertensive use, n%				0.369				**0.019**				0.369
Yes	2483 (82.90)	1.39 (1.12 ~ 1.71)	**0.002**		2483 (82.90)	1.16 (0.96 ~ 1.39)	0.121		2483 (82.90)	1.24 (1.02 ~ 1.52)	**0.035**	
No	512 (17.10)	1.80 (0.96 ~ 3.41)	0.069		512 (17.10)	0.49 (0.22 ~ 1.09)	0.081		512 (17.10)	1.15 (0.48 ~ 2.75)	0.752	
Statin use				0.092				0.681				0.873
Yes	1442 (42.69)	1.28 (1.00 ~ 1.65)	0.051		1442 (42.69)	1.08 (0.81 ~ 1.43)	0.603		1442 (42.69)	1.12 (0.83 ~ 1.50)	0.467	
No	1936 (57.31)	0.90 (0.47 ~ 1.76)	0.765		1936 (57.31)	1.10 (0.82 ~ 1.48)	0.511		1936 (57.31)	1.22 (0.83 ~ 1.78)	0.306	
Antihyperglycemic agent use, n%				0.524				0.162				0.256
Yes	859 (55.06)	1.41 (0.93 ~ 2.14)	0.102		859 (55.06)	0.81 (0.55 ~ 1.20)	0.287		859 (55.06)	0.91 (0.58 ~ 1.43)	0.675	

No	701 (44.94)	1.28 (0.77 ~ 2.13)	0.345		701 (44.94)	1.56 (1.18 ~ 2.08)	**0.002**		701 (44.94)	1.62 (1.17 ~ 2.26)	**0.004**	
KDM-BA				0.008				**0.013**				**0.03**
＜5	2570 (35.01)	0.94 (0.70 ~ 1.27)	0.698		2570 (35.01)	0.73 (0.47 ~ 1.14)	0.17		2570 (35.01)	0.80 (0.54 ~ 1.19)	0.275	
≥5	4770 (64.99)	1.43 (1.17 ~ 1.75)	**<.001**		4770 (64.99)	1.35 (1.14 ~ 1.60)	**<.001**		4770 (64.99)	1.47 (1.23 ~ 1.75)	**<.001**	

Abbreviations: HR, hazard ratio; CI, confidence interval; KDM-BA, Klemera–Doubal biological age acceleration; TyG, triglyceride-glucose index; TyG-BMI, TyG combined with BMI; TyG-WHtR, TyG combined with waist-to-height ratio.

Weighted Cox proportional hazards models, stratified by survival status and adjusted for age, Liverdisease, Cancer, Gender, Race, Education, Marital status, PIR, Smoking status, Drinking, CVD, CKD, ALT, AST, BUN, UA. Data are presented as HR (95% CI).

Table S10 Subgroup Analysis of Adjusted HRs for All-Cause Mortality Associated with TyG-related Indices in The PA Population

Variables	TyG			P for interaction	TyG-BMI			P for interaction	TyG-WHtR			P for interaction
	n (%)	HR (95% CI)	*P*		n (%)	HR (95% CI)	*P*		n (%)	HR (95% CI)	*P*	
**All-cause mortality**												
Age				**<.001**				**0.012**				**0.007**
＜65	5126 (65.39)	1.35 (1.18 ~ 1.54)	**<.001**		5126 (65.39)	1.17 (0.98 ~ 1.40)	0.08		5126 (65.39)	1.30 (1.08 ~ 1.56)	**0.006**	
≥65	2713 (34.61)	1.07 (0.99 ~ 1.17)	0.095		2713 (34.61)	0.97 (0.88 ~ 1.06)	0.505		2713 (34.61)	1.03 (0.94 ~ 1.13)	0.476	
Antihypertensive use				0.34				0.394				0.475
Yes	2581 (86.73)	1.16 (1.03 ~ 1.30)	**0.012**		2581 (86.73)	1.01 (0.90 ~ 1.13)	0.874		2581 (86.73)	1.11 (0.99 ~ 1.25)	0.069	
No	395 (13.27)	1.26 (0.90 ~ 1.75)	0.176		395 (13.27)	1.62 (0.82 ~ 3.23)	0.167		395 (13.27)	1.52 (0.82 ~ 2.80)	0.185	
Statin use				0.698				0.279				0.609
Yes	1766 (51.74)	1.29 (1.11 ~ 1.48)	**<.001**		1766 (51.74)	1.19 (1.01 ~ 1.41)	**0.04**		1766 (51.74)	1.25 (1.07 ~ 1.45)	**0.004**	
No	1647 (48.26)	1.23 (1.02 ~ 1.48)	0.027		1647 (48.26)	1.05 (0.82 ~ 1.34)	0.708		1647 (48.26)	1.15 (0.92 ~ 1.43)	0.219	
Antihyperglycemic agent use, n%				0.145				0.324				0.338
Yes	976 (56.45)	1.19 (1.06 ~ 1.34)	**0.004**		976 (56.45)	1.28 (1.02 ~ 1.61)	**0.034**		976 (56.45)	1.36 (1.09 ~ 1.70)	**0.007**	
No	753 (43.55)	1.42 (1.10 ~ 1.84)	**0.007**		753 (43.55)	0.93 (0.71 ~ 1.20)	0.562		753 (43.55)	1.01 (0.76 ~ 1.34)	0.958	
PA				0.803				0.573				0.692
＜5	3087 (39.38)	1.12 (0.97 ~ 1.30)	0.112		3087 (39.38)	0.98 (0.84 ~ 1.15)	0.831		3087 (39.38)	1.04 (0.90 ~ 1.22)	0.575	
≥5	4752 (60.62)	1.15 (1.05 ~ 1.26)	0.004		4752 (60.62)	1.01 (0.89 ~ 1.14)	0.868		4752	1.09 (0.96 ~ 1.22)	0.171	

									(60.62)			
**Cardiovascular mortality**												
Age custom				**0.005**				**0.014**				**0.003**
＜65	5126 (65.39)	1.37 (1.09 ~ 1.73)	**0.007**		5126 (65.39)	1.44 (1.09 ~ 1.89)	**0.009**		5126 (65.39)	1.62 (1.21 ~ 2.17)	**0.001**	
≥65	2713 (34.61)	1.07 (0.90 ~ 1.27)	0.45		2713 (34.61)	1.09 (0.87 ~ 1.36)	0.453		2713 (34.61)	1.14 (0.92 ~ 1.41)	0.219	
Antihypertensive use				0.126				0.674				0.508
Yes	2581 (86.73)	1.22 (0.99 ~ 1.50)	0.068		2581 (86.73)	1.14 (0.90 ~ 1.44)	0.269		2581 (86.73)	1.23 (0.97 ~ 1.56)	0.09	
No	395 (13.27)	0.29 (0.09 ~ 0.88)	**0.028**		395 (13.27)	0.56 (0.34 ~ 0.91)	**0.021**		395 (13.27)	0.38 (0.22 ~ 0.65)	**<.001**	
Statin use				0.136				0.052				0.443
Yes	1766 (51.74)	1.38 (1.11 ~ 1.72)	**0.003**		1766 (51.74)	1.45 (1.07 ~ 1.97)	**0.015**		1766 (51.74)	1.51 (1.13 ~ 2.03)	**0.006**	
No	1647 (48.26)	1.18 (0.77 ~ 1.82)	0.453		1647 (48.26)	1.08 (0.71 ~ 1.63)	0.725		1647 (48.26)	1.27 (0.84 ~ 1.91)	0.266	
Antihyperglycemic agent use, n%				0.285				0.565				0.857
Yes	976 (56.45)	1.34 (1.08 ~ 1.65)	**0.007**		976 (56.45)	1.00 (0.69 ~ 1.46)	0.986		976 (56.45)	1.09 (0.77 ~ 1.55)	0.633	
No	753 (43.55)	0.90 (0.65 ~ 1.25)	0.528		753 (43.55)	1.18 (0.76 ~ 1.82)	0.464		753 (43.55)	1.18 (0.75 ~ 1.86)	0.483	
PA				0.499				0.556				0.879
＜5	3087 (39.38)	1.04 (0.81 ~ 1.35)	0.752		3087 (39.38)	1.25 (0.97 ~ 1.61)	0.082		3087 (39.38)	1.20 (0.92 ~ 1.57)	0.178	
≥5	4752 (60.62)	1.24 (1.02 ~ 1.51)	**0.034**		4752 (60.62)	1.11 (0.85 ~ 1.46)	0.429		4752 (60.62)	1.25 (0.96 ~ 1.63)	0.093	

Abbreviations: HR, hazard ratio; CI, confidence interval; KDM-BA, Klemera–Doubal biological age acceleration; TyG, triglyceride-glucose index; TyG-BMI, TyG combined with BMI; TyG-WHtR, TyG combined with waist-to-height ratio.

Weighted Cox proportional hazards models, stratified by survival status and adjusted for age, Liverdisease, Cancer, Gender, Race, Education, Marital status, PIR, Smoking status, Drinking, CVD, CKD, ALT, AST, BUN, UA. Data are presented as HR (95% CI).
